# The Role of Chemotherapy in Management of Inoperable, Metastatic and/or Recurrent Melanotic Neuroectodermal Tumor of Infancy—Own Experience and Systematic Review

**DOI:** 10.3390/cancers13153872

**Published:** 2021-07-31

**Authors:** Małgorzata Styczewska, Małgorzata A. Krawczyk, Ines B. Brecht, Konrad Haug, Ewa Iżycka-Świeszewska, Jan Godziński, Anna Raciborska, Marek Ussowicz, Wojciech Kukwa, Natalia Cwalina, Emil Lundstrom, Ewa Bień

**Affiliations:** 1 The English Division Pediatric Oncology Scientific Circle, Medical University of Gdansk, 80-210 Gdansk, Poland; ncwalina@gmail.com (N.C.); emil.lundstrom@vgregion.se (E.L.); 2 Department of Pediatrics, Hematology and Oncology, Medical University of Gdansk, 80-210 Gdansk, Poland; mkrawczyk@gumed.edu.pl; 3 Department of Pediatric Hematology and Oncology, University of Tübingen, 72076 Tübingen, Germany; ines.brecht@med.uni-tuebingen.de (I.B.B.); konrad.haug@med.uni-tuebingen.de (K.H.); 4 Department of Pathology and Neuropathology, Medical University of Gdansk, 80-210 Gdansk, Poland; ewa.izycka-swieszewska@gumed.edu.pl; 5 Department of Pediatric Surgery, Marciniak Hospital, 54-049 Wroclaw, Poland; jgodzin@wp.pl; 6 Department of Pediatric Traumatology and Emergency Medicine, Wroclaw Medical University, 50-345 Wroclaw, Poland; 7 Department of Oncology and Surgical Oncology for Children and Youth, Institute of Mother and Child, 01-211 Warsaw, Poland; anna.raciborska@hoga.pl; 8 Department of Pediatric Hematology, Oncology and Bone Marrow Transplantation, Wroclaw Medical University, 50-556 Wroclaw, Poland; marek.ussowicz@umed.wroc.pl; 9 Department of Otorhinolaryngology, Faculty of Medicine and Dentistry, Medical University of Warsaw, 00-739 Warsaw, Poland; wojciechkukwa@gmail.com

**Keywords:** melanotic neuroectodermal tumor of infancy, inoperable, metastatic, recurrent, chemotherapy, adjuvant, neoadjuvant, systemic treatment

## Abstract

**Simple Summary:**

Melanotic Neuroectodermal Tumor of Infancy (MNTI) is a very rare neoplasm that most commonly develops within maxilla in infants. It usually has a benign clinical course and is treated with only surgery. However, patients with large, inoperable, metastatic or multiply recurring MNTI may require systemic treatment. The role of pre- and post-surgery chemotherapy (CHT) in the management of MNTI is unclear. Here, we have presented the disease courses and outcomes of four infants treated with multidrug CHT due to inoperable/recurrent MNTI. Additionally, a systematic literature review was performed which revealed 38 similar cases in the last 42 years. Most children with primarily inoperable MNTI responded to CHT, which allowed physicians to perform complete, non-mutilating delayed surgery. However, it is still uncertain whether CHT administered after incomplete resection of MNTI prevents recurrence. This study aimed to contribute to the establishment of standards of management in patients with inoperable, metastatic or persistently recurring MNTIs, which are currently lacking.

**Abstract:**

Melanotic Neuroectodermal Tumor of Infancy (MNTI) is a very rare pediatric neoplasm of neural crest origin. In most cases, it develops in infants as a localized tumor of the maxilla, and surgery is usually curative. In less than 10% of patients with inoperable, metastatic or persistently recurring MNTI, chemotherapy (CHT) may be considered; however, its role is still unclear. The aim of our study was to assess the efficacy of CHT in children with large, inoperable, metastatic and/or recurrent MNTI. Four such infants, treated with CHT in Polish and German centers of pediatric oncology, were presented. Additionally, a systematic literature search of the PubMed/MEDLINE, Scopus and Web of Science databases was performed, yielding 38 similar cases within the last 42 years. Neoadjuvant CHT, based mainly on the protocols for neuroblastoma, was often effective, allowing for complete delayed surgery in most cases. However, the role of adjuvant CHT in preventing recurrences after incomplete resection of MNTI remains unclear. Disseminated inoperable MNTI was almost universally associated with poor response to CHT and unfavorable outcome. Further investigations to elaborate standards of management in patients with inoperable, metastatic or persistently recurring MNTIs are necessary to improve outcomes.

## 1. Introduction

Melanotic neuroectodermal tumor of infancy (MNTI) is a very rare neoplasm mainly affecting children in the first year of life, with a median age at presentation of 4.5 months [1]. The clinical manifestation of MNTI is usually a fast-growing, painless, non-ulcerated solid mass with blue or black discoloration [2]. The tumor is localized most often in the head and neck region, with maxilla (62.2%), skull (15.6%) and mandible (7.8%) being the most common anatomical sites [1]. Localizations of MNTI within epididymis, extremities, ovary and endolymphatic sac have also been reported [3,4,5,6,7,8,9,10].

For many years, the origin of the tumor remained unclear and different theories were proposed, including odontogenic, retinal, neuroepithelial and neural crest derivation of the neoplasm [11]. This uncertainty is reflected in the many different names that have been used to describe this entity, including melanotic progonoma, congenital melanocarcinoma, pigmented epulis, melanoameloblastoma, retinal anlage tumor, pigmented tumor of the jaw of infants and others. Histochemical, biochemical and ultrastructural studies performed in recent decades have confirmed that MNTI belongs to the group of neoplasms originating from neural crest cells [12,13,14].

MNTI consists of two different populations of cells, often arranged in nests, cords, sheets and pseudo-glandular structures, embedded in fibro-collagenous stroma. One of the cell types are relatively large epithelioid cells resembling melanocytes, usually staining positive for both epithelioid and selected melanocytic markers. The second are small, round blue undifferentiated cells with neuroepithelial phenotype [2,15,16,17].

WHO 2017 “Head & neck tumors classification” defines MNTI as a locally aggressive, rapidly growing tumor [18,19]. In localized disease, surgery is a mainstay of treatment and, if performed radically, it is usually curative. In case of a local recurrence, which occurs in 15–20% of cases, the recommended treatment strategy is the repeated complete excision of the tumor [20]. 

Several cases of malignant MNTI with aggressive clinical course and presence of distant metastases have been described in the literature [21,22,23,24]. In such cases, as well as in primarily inoperable or persistently recurring MNTI, the optimal treatment strategy is uncertain. Since radical excision without severe mutilation is usually not possible, and radiotherapy (RTX) should not be proposed to young infants, a systemic treatment has to be considered. However, due to the rarity of the disease, only a few guidelines regarding the clinical management of children with inoperable, metastatic, or recurrent MNTI have been established to date, and the role of chemotherapy (CHT) as a neoadjuvant or adjuvant treatment has yet to be fully determined [20]. 

In this work, we presented four infants with inoperable and/or recurrent MNTI requiring systemic treatment. A systematic review of the literature regarding all cases of MNTI treated with CHT was also performed to evaluate the efficacy of CHT in MNTI. 

## 2. Case Reports

### 2.1. Patient 1

**Figure 1:**
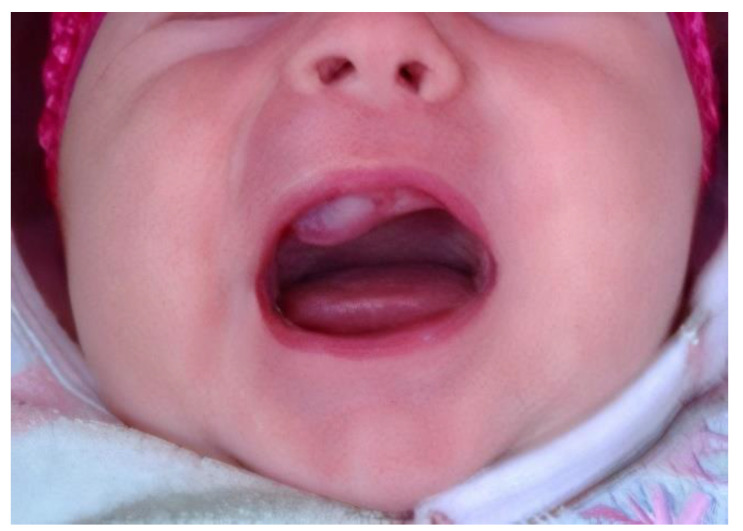
Patient 1: an intraoral tumor originating from the right side of the maxilla at first admission to the clinic.

**Figure 2:**
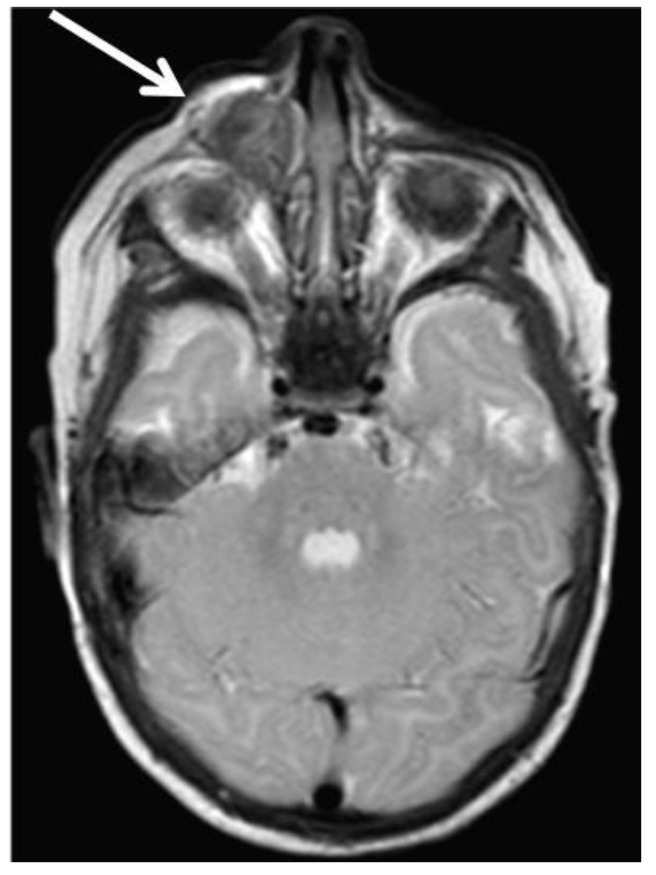
Patient 1: an axial T2-weighted MR scan shows well-defined pathological solid mass originating from the alveolar process of the right maxilla.

A 2-month-old female infant was admitted due to a fast-growing tumor of the right side of the maxilla, disfiguring the right cheek, nose and upper lip. The first symptoms occurred when the girl was 6 weeks old, when a tooth within the hard palate surrounded by a small, hard, non-painful bulge was found by the mother (Figure 1). It was diagnosed by a GP as a neonatal tooth and a decision was made to wait and see. However, during the next two weeks, the mass rapidly increased in size. Magnetic resonance (MR) displayed a pathological, well defined, solid mass measuring 23 × 22 × 18 mm, located within the alveolar process on the right side of the maxilla, reaching the midline. The bud of the right incisor was visible within the mass. The tumor bulged outside in the region of the nasal alar and invaginated into the mouth. No evidence of destruction of the hard palate, penetration into the orbital or nasal cavities and no evidence of enlarged lymph nodes (LN) were found (Figure 2). 

**Figure 3:**
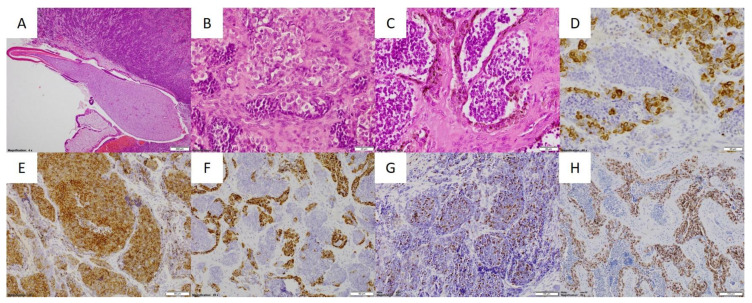
Patient 1: histopathological examination of MNTI. (**A**) Neoplastic tissue surrounding developing tooth with a root resorption (HE, 40×); (**B**) Tumor composed of small hyperchromatic and bigger epithelioid pale cells’ islands embedded in mesenchymal stroma (HE, 400×); (**C**) Nests of small neuroectodermal cells rimmed with brown melanocytic cells (HE, 400×); (**D**) Pan-cytokeratin expression in epithelial population (CKAE1.AE3, 400×); (**E**) Synaptophysin staining with different intensity (synaptophysin, 200×); (**F**) Melanocytic marker HMB-45 within the pigmented cells (HMB-45, 200×); (**G**) Proliferative index up to 15% (Ki-67, 200×); (**H**) SOX10 nuclear expression within the tumor (SOX10, 200×).

**Figure 4:**
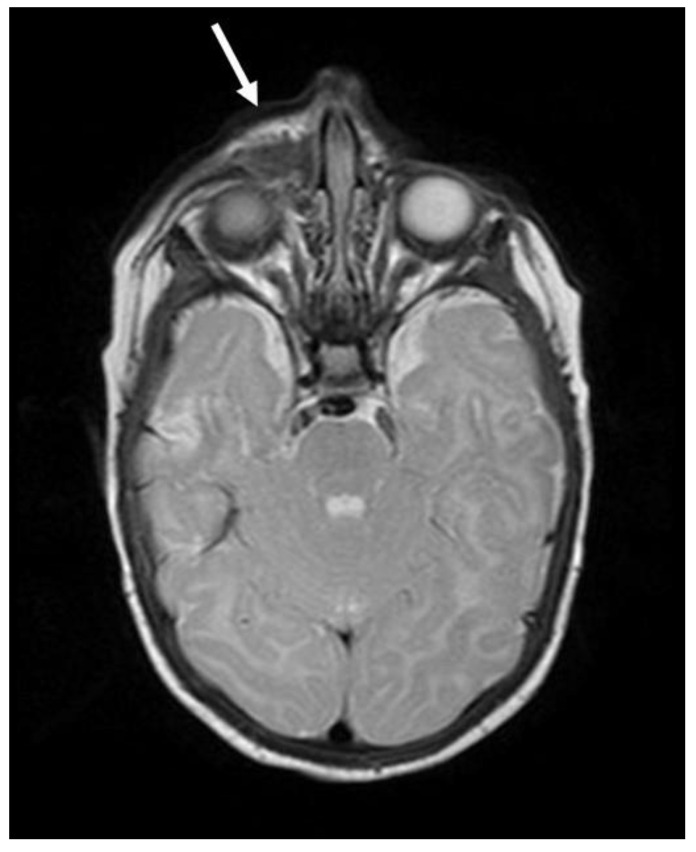
Patient 1: an axial T2-weighted MR scan shows recurrence of MNTI in the postoperative area.

The tumor was resected in the otolaryngology department; however, clear margins were not achieved. The histopathological examination revealed MNTI with typical morphology and immunophenotype (Figure 3). Three weeks after the surgery, a local recurrence was observed and the patient was referred to the department of Pediatric Oncology. MR revealed a mass measuring 16.5 × 16.5 × 22 mm visible in the postoperative area (Figure 4). The mass was invading the nasal concha and reaching the medial angle of the right eye; however, it did not penetrate into the orbit. Because the tumor was deemed unresectable without severe mutilation, a neoadjuvant CHT was introduced to reduce its size and extent. The patient received several courses of CHT according to the protocols for neuroblastoma (NBL) and sarcoma. The responses to particular courses of CHT varied (Table 1) but eventually, after eight courses, a partial regression of the tumor was achieved which enabled its complete non-mutilating resection. No recurrence was observed within 4.5 years post treatment. 

### 2.2. Patient 2

A 6-month-old girl was admitted due to visible facial asymmetry involving the left nasal region. The diagnostic imagings revealed the tumor within the left maxilla, which was then resected subtotally in a regional hospital. Based on histopathological examination, the diagnosis of MNTI was made. After two months, a local recurrence was diagnosed and the girl was referred to the department of Pediatric Oncology. The surgical excision of the relapse was performed but, once again, it proved incomplete. This time the histopathological assessment revealed malignant histological features of MNTI. One month after the surgery, the girl presented with the second local recurrence. She was qualified for treatment according to the protocol for skeletal Ewing sarcoma (ES) (Table 2). After six courses of VIDE, the tumor regressed substantially, which allowed for the delayed resection of the remaining mass. Unfortunately, the surgery did not ensure clear margins. Therefore, the child was given adjuvant CHT (8 courses of VAC) postoperatively. She finished therapy in complete remission of MNTI. However, seven months later, the girl was diagnosed with a second malignancy: acute lymphoblastic leukemia (ALL). She was treated according to the ALL IC-BFM 2009 protocol and was qualified for allogeneic hematopoietic stem cell transplantation (allo-HSCT). Currently, she is alive without evidence of disease with a follow-up of over 4 years after the end of ALL therapy. Genetic testing for germline pathogenic variants predisposing to cancer was not performed. 

### 2.3. Patient 3

**Figure 5:**
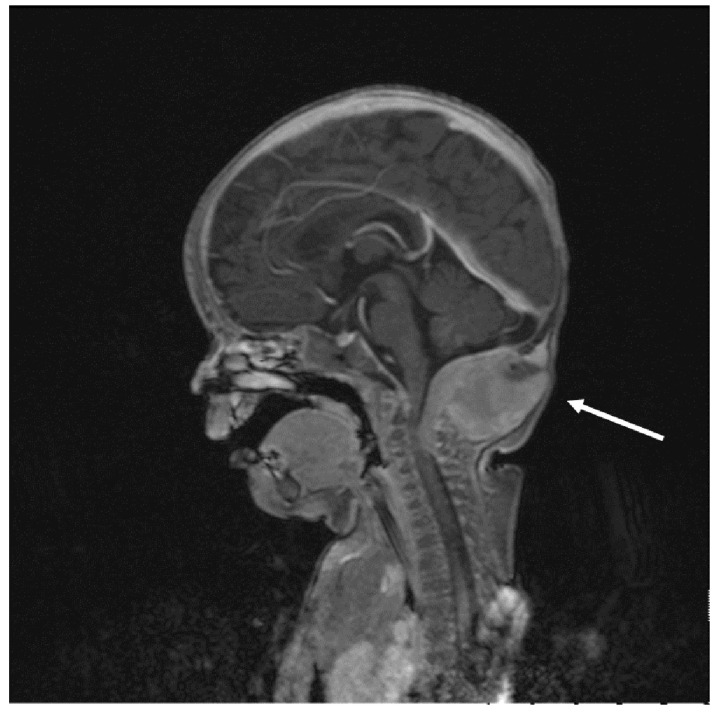
Patient 3: a sagittal T1-weighted MR scan shows large, suboccipital, mainly extracranial pathological solid mass compressing the spinal cord (C1–C3).

A 3-month-old female was admitted to the clinic due to an enlarging occipital tumor. The lesion was visible at birth, and the parents noticed that its size increased within the following weeks. During a routine checkup, a muscular weakness of the lower extremities was observed in the child, which led to CT and MR of the central nervous system (CNS). The imaging studies showed a suboccipital mass measuring 40 × 40 × 31 mm with epidural infiltration (Figure 5). It was compressing the spinal cord (C1-C3) and was located directly adjacent to the vertebral arteries. A biopsy followed by histopathological examination was performed, revealing the diagnosis of MNTI. Because of the rapid tumor growth, neoadjuvant CHT was started according to NBL protocol NB2004 (Table 3). In the second course of CHT, an accidental overdosage of doxorubicin happened and the next planned course of CHT was cancelled. Instead, a watch and wait policy was applied. The volume of the tumor was stable for three months, but then a slow progression occurred. Due to the high local tumor extension, only a partial resection was possible. The remaining mass was still compressing the spinal cord; thus, three further courses of CHT, not including anthracyclines, were administered. A follow-up echocardiography performed a year after treatment completion revealed anthracyclin-induced cardiomyopathy. The remaining tumor mass did not change in size for the next three years, but then a slow progression of the tumor was noted in the MR. Therefore, the current indication for RTX is under discussion.

### 2.4. Patient 4

A 4-month-old female was admitted due to a fast-growing tumor of the right maxilla, dislocating the nasal septum. An MR was performed, showing a solid 10 × 10 mm, well defined, slightly lobulated mass of the frontal medial palate with an embedded tooth. No infiltration of surrounding structures was detected. The diagnosis of MNTI was made based on the histopathological examination of the material from tumor biopsy. Subsequently, the resection of the tumor was performed, which was found microscopically incomplete. Three months later, the tumor recurrence was diagnosed. It was not feasible for resection, so CHT according to the NBL protocol NB2004 was started. After four courses of treatment with vincristine, cyclophosphamide and doxorubicin, the tumor size stabilized (Table 4). A second resection was performed; however, again, the tumor was not excised completely. Since then, no disease progression has been stated for over 1.5 years. 

## 3. Review of the Literature

### 3.1. Methodology

**Figure 6:**
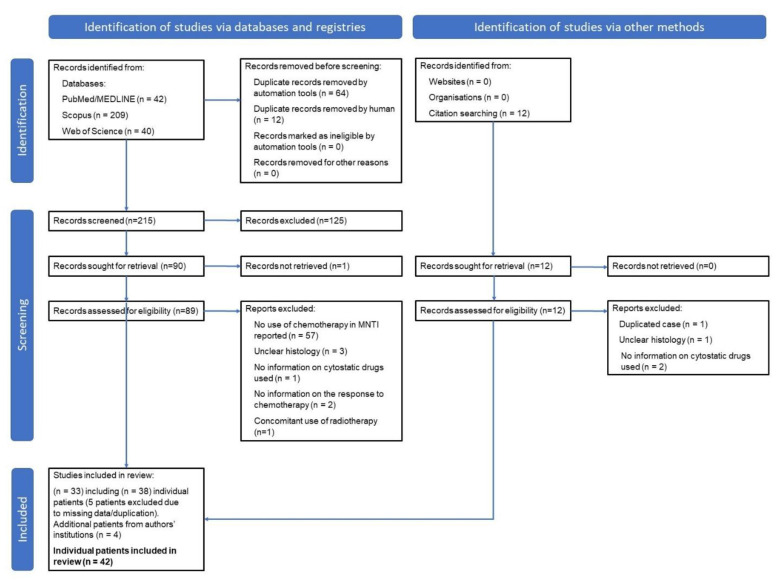
Flowchart of the study selection process (according to PRISMA 2020 guidelines).

The systematic literature review was performed according to the Preferred Reporting Items for Systematic reviews and Meta-Analyses (PRISMA) 2020 guidelines by searching the publications listed in the PubMed/MEDLINE, Scopus and Web of Science databases up to 26 April 2021 (Figure 6). The systematic search was performed by one reviewer (M.S.). Independently, the publications were searched by N.C. and E.L. (searched up until 2016) and M.K. (searched 2017–2021). Subsequently, all papers were verified by senior author (E.B.). Duplicated publications were removed with the use of automatic tool (Mendeley). Keyword search terms included: “melanotic neuroectodermal tumor of infancy”, “retinal anlage tumor” and “melanotic progonoma” combined with terms “chemotherapy” and “systemic treatment”. No additional search filters were applied. Additionally, the cases of children with MNTI treated with CHT were selected manually from the four main reviews [1,25,26,27]. The reference lists inserted in all publications were also searched for additional cases. Reports published in a language other than English were excluded. The data retrieved from each article included: age and sex of the patient, primary tumor site and size, presence of metastases, course of treatment, details of CHT treatment, follow-up and outcome. The demographic data of the patients were carefully analyzed to avoid duplication of cases. The series from literature was completed by four cases registered in the German Pediatric Rare Tumor Registry (STEP-Registry) and the Polish Pediatric Rare Tumors Database. Written informed consent was received from all parents at the time of registration.

Neoadjuvant CHT was defined as a CHT introduced (either in the first-line treatment or in the treatment of relapse) with the aim of diminishing an inoperable tumor and enabling delayed surgery. Adjuvant CHT was defined as a CHT introduced after tumor resection to treat residual tumor mass/avoid recurrence.

### 3.2. Results

The literature search yielded 54 children with MNTI treated with CHT. Among them, two cases were duplicates [16,28] and four cases were excluded due to unclear histology [29,30,31,32]. Eight cases were excluded due to lack of information on the cytostatic drugs administered to patients [16,33,34,35,36] or no data on the response to CHT [37,38]. In two patients, CHT was combined with RTX; in those cases, therefore, the response to CHT was impossible to define [23,39]. Eventually, 42 cases were analyzed, including 38 cases previously published in the literature and four cases of our own. CHT was given in all cases, either as a first-line treatment (n = 24) or as a treatment after relapse (n = 18). In each patient, the particular neoadjuvant/adjuvant CHT was analyzed only if data regarding the response to CHT were available. 

The demographic data of patients showed that the median age at diagnosis was 4 months. The anatomical locations of the tumors were: maxilla (n = 19; 45.2%), skull (n = 9; 21.4%), mandible (n = 5; 11.9%), femur (n = 2; 4.8%), CNS (n = 2; 4.8%), orbit (n = 2; 4.8%), epididymis (n = 1; 2.4%), suboccipital area wih an infiltration of the spinal canal (n = 1; 2.4%) and soft tissue of forearm (n = 1; 2.4%). Among 39 cases with known gender, a clear male predilection was found (the male to female ratio was 27:12; 69.2%:30.8%) (Table 5).

#### 3.2.1. Chemotherapy in the First-Line Treatment of MNTI

Chemotherapy was used as a component of the first-line treatment in 24 patients with MNTI, including one of our four patients (Table 6, Supplementary Materials Table S1). This subgroup consisted of 14 males and 7 females (in 3 patients, no data on gender was reported) aged between 1 day and 4 years (median age 4 months). The maximal tumor diameter ranged from 2.3 to 20.5 cm with a median size of 6 cm. The most commonly used CHT regimes included cyclophosphamide, doxorubicin, vincristine, ifosfamide, etoposide and cisplatin (Supplementary Materials Table S1).

##### Adjuvant CHT in the First-Line Treatment of MNTI

Ten patients with MNTI received adjuvant CHT postoperatively. In eight patients, the preceding surgery was incomplete. In seven, it was a resection of a localized tumor and in one patient, it was a resection of a metastatic focus [21,40,41,42,43,44,45]. Adjuvant CHT was also given to two children with regional disease after complete resections of the primary tumors and involved lymph nodes [22,46]. 

Among eight patients who received CHT after incomplete surgery, response to CHT was assessable in six. Complete response was noted in two patients, both with MNTI of the skull. Their CHT regimes consisted of either alternate courses of cyclophosphamide and doxorubicin [42] or the 8-in-1 protocol for medulloblastoma [41]. In two children, partial responses were obtained. In a child with localized MNTI of the skull, it allowed for the second-look complete surgery [43], while in a patient with metastatic disease, the partial response to CHT only lasted for a short time and was followed by PD [21]. Two children, including one of our patients (number 3), achieved stabilization of disease; however, progression occurred thereafter [40]. 

Two children in whom CHT followed complete resection of the tumor and involved lymph nodes and two children who received adjuvant CHT after R1 resection are alive without recurrences [22,44,45,46]. 

Altogether, seven of ten patients treated with adjuvant CHT did not experience recurrence or PD, with a follow-up ranging from 10 months to 4.5 years. 

##### Neoadjuvant CHT in the First-Line Treatment of MNTI

In 12 patients with inoperable and/or metastatic MNTI at diagnosis, the neoadjuvant CHT was introduced. The diagnosis was set based on a biopsy of the primary tumor (11 patients) or of an involved lymph node (one patient). 

In eight patients, partial responses to CHT were achieved, followed by delayed tumor resections [4,43,47,48,49,50,51,52]. The surgery was complete in five patients, of whom three are alive; one was lost to follow-up after late recurrence and one died due to acute cardiomyopathy. Incomplete delayed resections were performed in two children, of whom one survived. In one patient, the completeness of resection was not defined and the follow-up was not provided [52]. 

Four of 12 patients did not respond well to neoadjuvant CHT (stable disease in three, PD in one patient), however, in all of those cases, delayed surgeries were attempted [3,53,54]. Three patients survived, and one is currently being treated for late tumor recurrence. 

In total, 6 of 11 patients treated with neoadjuvant CHT due to inoperable and/or metastatic MNTI at diagnosis were disease-free at the time of publication [4,47,48,50,53,54]. One patient is alive with disease (our patient, number 3), two patients died (including one due to acute therapy-related complications) [49,51] and three patients were lost to follow-up [3,43,52].

##### CHT as the Only First-Line Treatment of MNTI

In three patients with inoperable MNTI, no tumor resection was performed. In two of them, the disease was successfully controlled by CHT only. In one child, a subtotal calcification of the tumor was observed during 10 months of multidrug CHT. One year after CHT cessation, no progression occurred [55]. In another child, CHT—consisting of etoposide and carboplatin—produced partial response with no regrowth within the next 16 months [16]. Conversely, in one patient with a huge inoperable MNTI of the maxilla, the response to one course of CHT composed of vincristine, cyclophosphamide and dactinomycin was poor (10% regression). The parents refused further treatment and the patient was lost to follow-up [56]. 

#### 3.2.2. Chemotherapy in the Treatment of Recurrence of MNTI

There were 18 patients with recurrence of MNTI who were treated with CHT (Table 7, Supplementary Materials Table S1). This group included three of our patients (numbers 1, 2 and 4). In all children, the recurrence developed after the first-line therapy composed of surgical tumor excision. In most publications, the completeness of resection was not specified; however, at least seven patients initially had only curettage or partial resection. The median time from initial surgery to the first relapse was 1.5 months (range: 2 weeks–18 months), with 16 local, one regional and one metastatic recurrence. Chemotherapy was administered as a treatment of the first, second and third relapses in 13, three and two patients, respectively. 

The CHT regimes to treat MNTI recurrence comprised mainly cyclophosphamide, doxorubicin, vincristine, etoposide and carboplatin (Supplementary Materials Table S2). 

##### Adjuvant CHT in the Treatment of Recurrence of MNTI

Adjuvant CHT was used in 12 patients with recurrent MNTI; in nine cases, after incomplete tumor excision (R1 in five, R2 in four), and in three patients with unspecified completeness of excision of the recurrence. The response to CHT was assessable in six children. One of them achieved long-term complete response [57], two had partial response after five and six months of CHT, respectively [24,43] and one patient achieved stabilization of disease [39]. In two children, disease progression was stated during CHT [53,58]. 

In six patients, including our patient number 2, the efficacy of CHT was impossible to evaluate as the CHT followed R1 or subtotal resections with no residual mass visible in imaging studies. However, in all of them, no recurrence was stated and the children have no evidence of disease with follow-ups ranging from 1 to 17 years [36,59,60]. 

CHT was given after resection of the first or second recurrence in nine and three children, respectively. All but one of the patients who received adjuvant CHT in the treatment of the first relapse had favorable outcomes [36,39,43,57,59,60]. At the time of publication, six of these children (four R1, one R2, one not specified) were alive and free of disease, one patient with residual disease remained stable and one child with partial response was still in treatment. One patient who developed metastatic relapse did not respond to CHT and was lost to follow-up with disseminated and progressive tumor [53]. 

Among three patients who developed two relapses each and received adjuvant CHT after resection of the second recurrence, one did not respond to CHT and died of PD [58]. Another patient had a partial response; however, progression of disease was stated 5 months after cessation of CHT. After further CHT and RTX, at the time of publication, this child was alive with disease [24]. The third patient (our patient number 2), however, was successfully cured of the second recurrence of maxillary MNTI. She received neoadjuvant CHT before the resection of the recurrence followed by adjuvant CHT. However, due to the second malignancy (ALL) which was diagnosed seven months after the completed MNTI treatment, she underwent another oncological therapy consisting of CHT and allo-HSCT. She has remained disease free with a follow-up of 6.5 years.

##### Neoadjuvant CHT in the Treatment of Recurrence of MNTI

Eight patients were reported to start therapy of relapse with CHT. It was the first, second and third recurrence in five, one and two patients, respectively. Six patients presented with local, one with regional and one with metastatic relapse.

Among six children with local relapses, one patient achieved complete response to VECI protocol adapted from NBL treatment [61]. The partial response to CHT was stated in three children, including two presented in our study. All of them underwent successful tumor resection and remained disease free [17]. In one child (our patient number 4), CHT followed by incomplete tumor resection led to long-term disease stabilization. One patient developed PD during neoadjuvant CHT. Nevertheless, complete resection of the tumor was possible [62]. All these children were alive at the time of publication, with follow-up periods ranging from 6 months to 14 years.

Among two patients with regional/metastatic relapses, one child did not respond to CHT. The other achieved temporary remission after CHT, but both eventually died due to PD [53,63].

## 4. Discussion

The optimal management of children with repeatedly relapsing, inoperable and/or disseminated MNTI has not yet been determined, due to the extreme rarity of this subgroup of MNTI. The Children’s Cancer & Leukaemia Group (CCLG) Guidelines published in 2004 suggested that, in such cases, systemic CHT would be an option [20]. In this paper, based on literature review and our own experiences, we aimed to determine the incidence of MNTIs requiring systemic treatment and analyze the efficacy of the most frequently used CHT regimes.

We found 54 reported cases of MNTI treated with CHT. For 38 patients, sufficient data on cytostatic drugs, response to CHT and disease course was available. Four cases of MNTI treated in oncological centers in Poland and Germany were added, resulting in 42 cases feasible for analysis. Three out of our four cases developed in the maxilla; however, nearly 55% of the studied group had extramaxillary tumors. This was significantly more frequent in comparison to children with localized MNTI, treated with surgery and/or RTX, as reported by Rachidi et al. (34%). It seems that aggressive MNTIs tend to develop in sites other than the maxilla. Additionally, the significant male predilection observed in patients treated with CHT (M:F 69.2%:30.8%) stands in contrary to the almost equal gender distribution reported in patients with localized MNTIs (M:F 57%:43%) [1].

In 2004, the CCLG proposed two CHT protocols for inoperable MNTI. Their regimen 1 consists of cyclophosphamide and vincristine and was proposed for management of tumor recurrence occurring despite two surgical interventions. The more aggressive regimen 2 is intended for therapy of inoperable or metastatic disease at diagnosis or in case of no response to regimen 1. It was adapted from the protocol OPEC/OJEC for stage 4 NBL and includes vincristine (O), cisplatin (P), etoposide (E), cyclophosphamide (C) and carboplatin (J) [20]. However, these protocols have not been widely used. Few authors reported successful use of regimen 1 [36,44] and only two cases of MNTI treated with adjuvant OPEC/OJEC protocol after incomplete resection have been published since 2004 [36]. Although both latter patients survived, the effectiveness of these regimens is difficult to assess. Various other CHT protocols have been used for inoperable/metastatic MNTI in the last 15 years [22,24,39,45,50,51,54].

Including the aforementioned patients treated according to CCLG guidelines, 12 out of 39 reported cases of inoperable/recurrent and/or metastatic MNTI were treated with protocols directly adapted from NBL treatment [36,44,45,54,60,61,62]. Within this group are three of our patients, among whom one (patient number 1) responded to a regime consisting of vincristine, cyclophosphamide and carboplatin with etoposide, successfully used in infant NBL. Two other children (patients 3 and 4) achieved stabilization of the disease following NB2004 regimen.

The reason for choosing regimes adapted from NBL treatment is the neural crest origin of both MNTI and NBL. Neven at al. reported a 1p deletion and gain of chromosome 7q in one child with MNTI, indicating the genetic analogy to NBL [61]. In a subset of children with MNTI, elevated urine concentrations of vanillylmandelic acid (VMA) and homovanillic acid (HVA) have been observed, similarly to NBL [21,22,51]. Moreover, the component of neuroblast-like cells within MNTI has been suggested to be responsible for the aggressiveness of the tumor. In some cases of MNTI, the small, round blue component significantly predominated over epithelioid cells in metastatic lesions, whereas in the primary tumor, the two components were balanced. [46,58]. In cases evaluated histologically both before and after CHT, the CHT resulted in almost complete reduction of the small round blue cell component of MNTI; however, the reduction of epithelioid cells was poor [4,62].

Accordingly, the efficacy of NBL-derived protocols in MNTI treatment seems to be satisfactory. Notably, all six patients treated with adjuvant CHT after R1 resections (two in first-line treatment, four in relapsed tumor) experienced no recurrence [36,44,45,60]. The neoadjuvant CHT adapted from NBL treatment was used in the treatment of primary tumor and relapses in two and four patients, respectively. The responses varied; however, all patients were alive at the time of publication (all but one underwent delayed surgery) [54,61,62].

In our patient number 2, a CHT protocol for ES was successfully used as both neoadjuvant and adjuvant treatment of recurrent maxillary MNTI. In five other children, the name of the regimen was not mentioned, but the chemotherapeutic combinations were probably also adapted from ES treatment [22,50,55,56,57]. The cellular origin of ES has not been fully determined. Some authors have suggested neural crest derivation similar to MNTI [64]. Consequently, several MNTI cases were found to express CD99, a marker used in diagnosis of ES group tumors [3,45,65,66]. In a series of children with MNTI, CD99 was expressed in one out of eight tumors and was associated with malignant behavior [15].

In our analysis, the efficacy of MNTI treatment based on ES regimes was difficult to assess due to the small number of cases. Excluding our aforementioned patient, two other children were treated with adjuvant CHT. One of them had no recurrence after R0 resection of primary tumor and metastatic LNs [22]. Another child treated with CHT after R2 surgery of recurrent MNTI demonstrated complete response [57]. Among three patients who underwent neoadjuvant CHT adapted from ES protocol in the first-line treatment, one achieved PR and two had stable disease [50,55,56]. Therefore, in general, the efficacy of regimes adapted from ES treatment seems to be acceptable in MNTI.

Some patients with MNTI were treated according to the protocols for other pediatric solid tumors. Creytens et al. reported the successful use of neoadjuvant CHT according to the RMS 2005 protocol, which resulted in a significant response allowing for delayed complete tumor resection [4]. Another patient had a partial response to a protocol for high-risk soft tissue sarcomas; however, progression occurred 5 months after the end of treatment [24]. Our patient number 1 also received two courses of CHT based on the Cooperativen Weichteilsarkom Studiengruppe (CWS)-2006 protocol for soft tissue sarcomas (I2VAdr and I2VE), which produced stabilization of tumor size. Two other children with MNTI of the pineal region and skull were treated according to the 8-in-1 protocol used in the treatment of malignant intracranial tumors. In one patient, after subtotal surgery, a complete response and no recurrence after 10 months were observed [41]. In another, the CHT followed partial resection and resulted in stabilization of the tumor, lasting for nine months after cessation of treatment [40]. Response of MNTI to CHT meant for other pediatric solid tumors may result from the use of similar cytostatic drugs (e.g., vincristine, doxorubicin) in these neoplasms.

In order to optimize therapeutic approaches to MNTI, several attempts were made to find genetic connections of MNTI to other malignancies. Gomes et al. revealed BRAFV600E mutation in one of three analyzed cases of MNTI which may indicate further options for targeted therapy [67]. Moreover, a case of fibular MNTI with a germline CDKN2A loss-of-function mutation was also described. Since inherited CDKN2A mutations are associated with familial melanoma syndromes and the epithelioid MNTI cells are supposed to be immature melanocytes, it was suggested that the genetic rearrangement might be partially responsible for the development of MNTI in this particular patient [66]. However, none of these discoveries has yet been translated into clinical management of the tumor.

Most children with inoperable/recurrent and/or metastatic MNTI analyzed in this study were treated with CHT administered preoperatively (neoadjuvant CHT) and/or after surgical attempts (adjuvant CHT).

In children with inoperable or incompletely resected primary MNTI or MNTI relapse, neoadjuvant CHT seems to be promising. In eight out of 12 children who underwent neoadjuvant CHT in the first-line treatment, partial tumor shrinkage was stated [4,43,47,48,49,50,51,52]. This enabled physicians to perform delayed surgery, which was complete in 5 out of 8. Moreover, two children were successfully treated for MNTI with CHT only. In these patients, the CHT was probably intended to be neoadjuvant but, eventually, the delayed surgeries were not performed [16,55]. Both patients were alive at the time of publication without further progression or recurrence. It seems, therefore, that in the subset of children with initially inoperable MNTI in whom complete or good partial response to CHT is observed, long-term remission may be achieved even without performing delayed resection. This approach may reduce possible mutilation; however, the risk of progression/recurrence after treatment with CHT only is difficult to determine.

Among eight children in whom neoadjuvant CHT was used as a first treatment of relapse, one patient achieved complete response to NBL VECI protocol [61], while four others (including two of our patients) achieved partial response. This allowed for delayed non-mutilating surgeries [17,53].

However, the final outcome did not always correlate with response to neoadjuvant CHT. After complete delayed surgery following good response to CHT of primary MNTI, 2 out of 5 patients died due to recurrence or complications [43,49]. Conversely, 2 out of 4 children who did not respond to CHT survived after delayed surgery, independent of its completeness [53,54]. In relapsed MNTI, complete delayed resection was achieved in a patient who did not respond to CHT [62].

Adjuvant CHT is usually the only available therapeutic option in patients with macroscopic tumor masses or metastases remaining after surgery, especially when reoperation is not possible. However, it seems to be beneficial only in a subset of cases. Adjuvant CHT following initial partial (R2) resection of MNTI was effective in 3 out of 6 patients [42,43,68]. Among four patients who underwent CHT after partial (R2) resection of relapse, one achieved complete response, one partial response, and one was still in treatment at the time of publication [24,43,57].

Most authors agree that adjuvant CHT might prevent recurrence in patients with microscopically positive surgical margins (R1), high mitotic rate, aneuploidy and unfavorable location of MNTI [21,44,45,49,60]. In our study, however, only seven such cases were reported (two in the first-line treatment and five after recurrence) [36,44,45,60]. Lack of visible tumor in imaging studies makes it impossible to assess the response to CHT. However, in all the children undergoing adjuvant CHT, no recurrence was noted after follow-ups of 1 to 17 years. On the other hand, the recurrence rate of MNTI does not clearly correlate with the radicality of surgery or the use of adjuvant chemotherapy [1,16]. There are numerous reports on uneventful courses of MNTI after R1 or subtotal surgery with no adjuvant treatment [16,36]. Moreover, several cases of spontaneous regression or calcification of the macroscopic residual MNTI were noted [48,54,69]. However, a recently published systematic review including 429 MNTIs of maxilla and mandible, suggests that the risk of recurrence increases when only curettage of the tumor is performed [18]. Therefore, it would be of great value to identify the subset of patients with high risk of relapse of MNTI after R1 surgery, which would benefit from adjuvant CHT. One significant factor predicting the risk of relapse is the patient’s age at diagnosis. In children diagnosed within the first 2 months of life, the recurrence rate is the highest, while in those beyond 4, 5 months of age it is minimal [1]. Most recurrences occur within the first 4 weeks after surgery and the risk of regrowth decreases with time, with only anecdotal reports of recurrences developing more than six months after completion of treatment [25]. In our study group, among 18 patients who were treated with CHT due to relapse of MNTI, the median age at diagnosis was 3 months, as compared to 4 months in the whole study group.

The localization of the primary MNTI may also be a risk factor for relapse. The recurrence rate was found to be higher in MNTIs affecting the mandible (33.3%) and the skull (31.8%), than in maxillary (19.3%) and intracranial (12.5%) tumors [25]. However, this tendency was not observed in the group of patients treated with CHT for recurrent MNTI, as the relapsed tumors were localized predominately in the maxilla (n = 13; 72.2%), skull (n = 3; 16.7%), mandible (n = 1; 5.6%) and orbit (n = 1; 5.6%).

No clear histological or biological markers of malignancy or recurrence in MNTI have been reported to date. Aneuploidy was suggested to be a negative prognostic factor, since two such cases reported by Pettinato et al. recurred within 1 month. However, the recurrences were observed in the diploid tumors as well [18,70]. Some authors implied that the presence of necrosis and mitotic figures may be associated with a malignant course of disease [53]. Still, these features have also been found in benign tumors [65]. Based on a single case, Barrett et al. suggested that a high Ki-67 proliferation marker and the presence of CD99 expression could indicate aggressive clinical behavior of MNTI [15]. However, the Ki-67 status assessed in 36 cases of maxillary/mandible MNTIs did not correlate with recurrence rate [18] and the CD99 expression was also reported in typical MNTIs, with benign histologies and outcomes [2,65].

Thus, in spite of various attempts, it is currently impossible to reliably assess the risk of recurrence based on clinical characteristics of MNTI. In our opinion, adjuvant CHT may be considered in children in whom the tumor has been removed incompletely, particularly in those within the first two months of life, when the recurrence risk is the highest and the use of radiotherapy is avoided. However, the potential short- and long-term adverse effects of cytostatic drugs in young infants should be carefully taken under consideration. Choi et al. reported the death of a patient with MNTI due to cardiomyopathy during treatment including doxorubicin [49]. Therefore, the risk-to-benefit ratio should always be carefully evaluated for each patient with MNTI before adjuvant CHT is given.

There seems to be a group of patients with MNTI who respond poorly to CHT, regardless of which cytostatic drugs and protocols are used. Two out of three patients in whom distant organ metastases were present at diagnosis or were detected during the course of treatment succumbed to the disease or were lost to follow up in PD [21,53]. The only patient with leptomeningeal dissemination of recurrent MNTI who was alive with disease three years following treatment responded to salvage RTX treatment [24]. Among four children with metastases limited to LN, the outcome was good in two patients who underwent complete excision of the main mass and involved LN, followed by adjuvant CHT [22,46]. However, two other patients in whom radical surgery was not feasible, died of disease despite long, multimodal treatment [51,63]. It suggests that the presence of inoperable disseminated disease is associated with poor response to CHT and, subsequently, with unfavorable outcomes.

The optimal follow-up regimen for patients with MNTI after surgical resection has not yet been established. In patients with maxillary or mandibular tumors, almost all local and regional recurrences were reported to occur within 6 months after resection. Therefore, it has been suggested to perform regular and frequent clinical and ultrasound examinations [1]: weekly in in the first month after surgery, every second week for the next 3 months and once a month for the following 6–12 months. It would also be important to perform MR of the resected tumor area every 3 months during this time period.

In patients with MNTI localized within the skull or CNS, relapses may occur later, even years after primary surgery [25]. Moreover, in the analyzed group, all three patients with distant metastases of MNTI had primary tumors located in the skull or CNS and developed intracranial metastases (in one case, disseminated via ventriculoperitoneal shunt) [21,24,53]. This may indicate a greater (but still very small) risk of dissemination in children with MNTI in these locations. Therefore, it may be suggested that patients with CNS/skull MNTI should undergo regular surveillance with MR of the head for at least 5 years after treatment. As no other locations of distant metastases of MNTI have been described in recent decades, it seems that routine follow-up imaging examinations of the chest, abdominal cavity and pelvis are not necessary.

In children with inoperable, metastatic and/or multiply relapsing MNTI who were treated with CHT—either neoadjuvant or adjuvant—the follow-up examinations should additionally include regular clinical, laboratory and imaging assessment of the renal, liver, eye, hearing and cardiac function. The risk of second malignancy should also be considered and properly addressed.

## 5. Conclusions

There are no established guidelines for optimal management in children with inoperable, metastatic or repeatedly recurrent MNTI. In such cases, CHT may be administered on a neoadjuvant and/or adjuvant basis. Considering the biological similarity of neuroblast-like components of MNTI and NBL, CHT adapted from NBL treatment protocols has been used most frequently with beneficial outcomes. Neoadjuvant CHT is usually effective, allowing physicians to perform delayed complete and non-mutilating surgery in a large subset of patients. Our review and own experiences indicate that CHT may be considered in a child with MNTI not feasible for initial tumor resection; however, significant awareness of severe complications is needed.

The role of adjuvant CHT in preventing recurrences after subtotal excision of MNTI remains unclear. The presence of inoperable disseminated disease is almost universally associated with poor response to CHT and unfavorable outcomes. Further investigations on the histological and molecular features of MNTI are required to adjust the treatment to the biology of the tumor and search for new targeted therapies. International collaborative studies with unified standards of management of children with inoperable, metastatic or persistently recurring MNTI and prospective enrollment in national or international databases are mandatory if we are to improve the knowledge and the prognosis of children with these rare types of MNTI.

## 6. Limitations of the Study

This retrospective study had several limitations. First, the analyzed group of patients is relatively small and heterogenous, so it is difficult to undoubtedly determine the exact efficacy of neoadjuvant and adjuvant CHT in the treatment of MNTI. Moreover, the definitions of response to CHT (partial response, stable disease, progression) have not been consistent and clearly defined between reports.

Additionally, some of the studies included in our analysis were published a few decades ago. Thus, we cannot exclude that, in a subset of analyzed patients, the tumor would nowadays be pathologically classified as a neoplasm other than MNTI.

## Figures and Tables

**Table 1:** Chemotherapy in patient 1.

Type of Treatment	Cytostatic Drugs	Number of Courses	Response
neoadjuvant	CADO (cyclophosphamide, vincristine, doxorubicin)	1	stable disease (tumor growth stopped)
neoadjuvant	VP-16 + Carbo (etoposide + carboplatin)	2	minor partial response → slow tumor progression
neoadjuvant	I2VAdr (ifosfamide, vincristine, doxorubicin with dose reduction to 3/4)	1	stable disease
neoadjuvant	I2VE (ifosfamide with dose reduction to 2/3, vincristine, etoposide)	1	stable disease
neoadjuvant	CADO (cyclophosphamide, vincristine, doxorubicin)	1	stable disease
neoadjuvant	VP-16 + Carbo (etoposide + carboplatin)	1	minor partial response
neoadjuvant	CO (vincristine, cyclophosphamide)	1	minor partial response

**Table 2:** Chemotherapy in patient 2.

Type of the Treatment	Cytostatic Drugs	Number of Courses	Response
neoadjuvant	VIDE (vincristine, ifosfamide, doxorubicin, etoposide)	6	partial response
microscopically incomplete (R1) resection of the tumor			
adjuvant	VAC (vincristine, dactinomycin cyclophosphamide)	8	no recurrence
ALL treatment (chemotherapy + allo-HSCT)	vincristine, daunorubicin, L-asparaginase, cyclophosphamide, ifosfamid, cytarabine, inthrathecal cytarabine, intrathecal metothrexate, doxorubicin, 6-mercaptopurin, 6-tioguanine, vindesine, etopozide	>2 years of treatment	no recurrence of ALL and MNTI

ALL = acute lymphoblastic leukemia; allo-HSCT = allogeneic hematopoietic stem cell transplantation.

**Table 3:** Chemotherapy in patient 3.

Type of the Treatment	Cytostatic Drugs	Number of Courses	Response
neoadjuvant	N4 (vincristine, cyclophosphamide, doxorubicin)	1	stable disease
neoadjuvant	N4 (vincristine, cyclophosphamide, doxorubicin—accidental overdosage)	1	stable disease→watch&wait→slow progression
partial resection of the tumor			
adjuvant	N5 (cisplatin, etoposide, vindesine)	1	not assessed
adjuvant	N6 (vincristine, dacarbazin, ifosfamide, without doxorubicin)	1	stable disease
adjuvant	N5 (cisplatin, etoposide, vindesine)	1	stable disease→follow-up →progession

**Table 4:** Chemotherapy in patient 4.

Type of the Treatment	Cytostatic Drugs	Number of Courses	Response
neoadjuvant	N4 (vincristine, cyclophosphamide, doxorubicin)	4	stable disease

**Table 5:** Demographic and clinical data of the analyzed patients.

All Patients	*n* = 42 (100%)
own cases	n = 4 (9.5%)
literature reports	n = 38 (90.5%)
Sex	
female	n = 12 (28.6%)
male	n = 27 (64.3%)
unknown	n = 3 (7.1%)
Median age at diagnosis (mo)	4 (range: 0–48)
Tumor site	
maxilla	n = 19 (45.2%)
skull	n = 9 (21.4%)
mandible	n = 5 (11.9%)
femur	n = 2 (4.8%)
CNS	n = 2 (4.8%)
orbit	n = 2 (4.8%)
epididymis	n = 1 (2.4%)
suboccipital area	n = 1 (2.4%)
forearm	n = 1 (2.4%)
Median tumor size at diagnosis (cm)	4 (range: 1–20.5)
Metastases at diagnosis	
no	n = 12 (28.6%)
yes	n = 4 (9.5%)
lymph nodes	n = 3 (7.1%)
distant metastases	n = 1 (2.4%)
unknown	n = 26 (61.9%)

CHT = chemotherapy; cm = centimeters; CNS = central nervous system; mo = months.

**Table 6:** Summary data of the patients receiving CHT in the first-line treatment of MNTI (n = 24).

Type of CHT	Number of Patients	Response to CHT	Outcome
adjuvant	10 (41.7%) *		
after macroscopically incomplete (R2) surgery of the primary tumor	5	CR: 2	NED: 2
		PR: 1	NED: 1
		SD: 2	AWD: 2*
after macroscopically incomplete (R2) surgery of the metastatic lesion	1	PR: 1	DOD: 1
after microscopically incomplete (R1) surgery of the primary tumor	2	NA: 2	NED: 2
after complete (R0) surgery of the primary tumor and involved LNs	2	NA: 2	NED: 2
neoadjuvant	12 (50%) *		
after biopsy of primary tumor	11	PR: 7	NED: 4 D of complications: 1 LFU: 2
		SD: 3	NED: 1 AWD: 1 * LFU: 1
		PD: 1	NED: 1
after biopsy of involved LN	1	PR: 1	DOD: 1
the only treatment	3 (12,5%) *		
after biopsy	3	PR: 2	AWD: 2 *
		SD: 1	LFU: 1
together	24 (100%)	CR: 2 PR: 12 SD: 4 SD; SD: 1 ** PD: 1 NA: 4	NED: 13 AWD: 4 * DOD: 2 D of complications: 1 LFU: 4

AWD = alive with disease; CHT = chemotherapy; CR = complete response; D = died; DOD = died of disease; LFU = lost to follow-up; LNs = lymph nodes; NA = not assessable; NED = no evidence of disease; PD = progressive disease; PR = partial response; SD = stable disease; * the numbers/percentage scores do not sum up to the summarized numbers/100% due to the overlap of the patients (details provided in the supplementary Table S1); ** patient who underwent both neoadjuvant and adjuvant CHT.

**Table 7:** Summary data of the patients receiving CHT in the treatment of recurrent MNTI (n = 18).

Type of CHT	Number of Patients	Response to CHT	Outcome
adjuvant	12 (66.7%) *		
after macroscopically incomplete (R2) surgery of the recurrent tumor	4	CR: 1	NED: 1 *
		PR: 2	AWD: 2
		SD: 1	AWD: 1
after microscopically incomplete (R1) surgery of the recurrent tumor	5	NA: 5	NED: 5*
after unspecified surgery of the recurrent tumor	3	PD: 2	DOD: 1 LFU: 1 (in progression) *
		NA: 1	NED: 1*
neoadjuvant	8 (44.4%) *		
after local recurrence	6	CR: 1	NED: 1*
		PR: 3	NED: 3*
		SD: 1	AWD: 1
		PD: 1	NED: 1*
after regional/metastatic recurrence	2	PR: 1	LFU: 1 (in progression) *
		PD: 1	DOD: 1
together	18 (100%)	CR: 2 PR: 2 PR; PD: 1 ** PR; NA: 1 ** SD: 2 PD: 4 NA: 6	NED: 11 * AWD: 4 DOD: 2 LFU: 1*

AWD = alive with disease; CHT = chemotherapy; CR = complete response; DOD = died of disease; LFU: lost to follow-up; LN = lymph nodes; NA = not assessable; NED = no evidence of disease; PD = progressive disease; SD = stable disease; * the numbers/percentage scores do not sum up to the summarized numbers/100% due to the overlap of the patients (details provided in the supplementary Table S2); ** patients who underwent both neoadjuvant and adjuvant CHT.

## Data Availability

The data presented in this study (additional information on four own patients) are available on request from the corresponding author. The data are not publicly available due to privacy of the patients. The other data presented in this study (cases of patients with MNTI found in reviewed articles) are openly available in public databases (PubMED/MEDLINE, Scopus, Web of Science).

## Supplementary Materials

The following are available online at https://www.mdpi.com/article/10.3390/cancers13153872/s1, Table S1: Detailed data of the patients treated with chemotherapy in the first-line treatment of MNTI; Table S2: Detailed data of the patients treated with chemotherapy in the recurrence of MNTI; Supplementary material S3: Systematic literature search protocol.
