# Supplementary material for: The Role of Chemotherapy in Management of Inoperable, Metastatic and/or Recurrent Melanotic Neuroectodermal Tumor of Infancy—Own Experience and Systematic Review
Source: Cancers (Basel). 2021 Jul 31;13(15):3872. doi: 10.3390/cancers13153872 (PMC8345623; doi:10.3390/cancers13153872)
Supplement: Supplementary file 1 [file cancers-13-03872-s001.zip › 30.07.21 MNTI Supplementary table S1 first-line treatment.pdf]

Table S1. Detailed data of the patients treated with chemotherapy in the first-line treatment of MNTI.

PATIENTS TREATED WITH ADJUVANT CHT

patient's no.	author, year of study	patient's age at diagnosis [months], sex	primary tumor site and size [cm]	metastases at diagnosis	initial management (before adjuvant CHT)	adjuvant CHT (cytostatic drugs, duration)	response to CHT	further course of treatment	follow-up from the beginning of treatment	outcome
1.	Cohen 1988 [1]	4, ND	CNS, ND	ND	R2 resection	**vincristine, lomustine, methylprednisolone, procarbazine, hydroxyurea, cisplatin, cytosine arabinoside, cyclophosphamide**[i] 12 months	stable disease	progression → RTX	26 months	alive with disease
2.	De Chiara 1992 [2]	17, M	epididymis 2.3x0.9	yes, LN	complete resection → LN resection	**cyclophosphamide, doxorubicin, cisplatin, etoposide**[ii] 18 months	no recurrence	-	28 months	NED
3.	Hoshino 1994 [3]	5, M	skull 4x4	no	R2 resection → R2 resection	**vincristine, lomustine, methylprednisolone, procarbazine, hydroxyurea, cisplatin, cytosine arabinoside, cyclophosphamide**[iii] 3 months	complete response	-	≈ 10 months	NED
4.	Patankar 1998 [4]	9, ND	skull ND	ND	biopsy → R2 resection	**cyclophosphamide, doxorubicin**[iv] 4 months	complete response	-	≈ 23 months	NED
5.	Kumari 2005 [5]	4, M	skull 9.2x7.8	ND	R2 resection	**vincristine**, **cyclophosphamide**[v] 6 months	partial response	R0 resection	16 months	NED
6.	Yoo 2014 [6]	28, F	CNS, ND	yes, spinal canal, inguinal sac peritoneum, cardiophrenic region (disseminated through VP shunt)	R2 resection of the inguinal mass	**ifosfamide, doxorubicin, vincristine**[vi] 1,5 months	partial response	progression	5 months	DOD
7.	Higashi 2016 [7]	3, F	maxilla, ND	ND	biopsy -> R1 resection	**vincristine, cyclophosphamide, pirarubicin, cyclophosphamide, carboplatin**[vii] ≈ 5 months	no recurrence	-	≈ 3 years	NED
8.	Murphy 2016 [8]	3, M	maxilla 3.5x2x1	no	biopsy → R1 resection → R1 resection	**vincristine**, **cyclophosphamide**[viii] 2,5 months	no recurrence	-	3 years	NED
9.	Azari 2016 [9]	6, M	mandible 9.4x6.7x6	yes, cervical LN	biopsy → R0 resection	**ifosfamide, vincristine, doxorubicin/dactinomycin**[ix] 7 months	no recurrence	-	4,5 years	NED
10.	current study, patient 3*	3, F	suboccipital area, spinal canal 4x4x3.1	no	biopsy → 2 courses of neoadjuvant CHT → R2 resection	**cisplatin, etoposide, vindesine**, **vincristine, dacarbazine, ifosfamide**[x] 2 months	stable disease	progression after 3 years	4,5 years	alive with disease

PATIENTS TREATED WITH NEOADJUVANT CHT

patient's no.	author, year of study	patient's age at diagnosis [mo], sex	primary tumor site and size [cm]	metastases at diagnosis	initial management (before neoadjuvant CHT)	neoadjuvant CHT (cytostatic drugs, duration)	response to CHT	further course of treatment	follow-up from the beginning of treatment	outcome
11.	Pierre-Kahn 1992 [10]	2, F	skull 10x6	ND	biopsy	**cyclophosphamide, doxorubicin**	stable disease	R0 resection	7 years	NED
12.	Mello 2000 [11]	6, F	maxilla 6	ND	biopsy	**vincristine, cyclophosphamide, doxorubicin, vincristine, cisplatin, tenoposide**[xi] 2,5 months	partial response	R0/R1 resection	≈ 22 months	NED
13.	Kumari 2005 [5]	4, F	skull 20.5x18.5	no	biopsy	**vincristine, doxorubicin, cyclophosphamide**[xii] 12 months	partial response	R0 resection	20 months	recurrence, lost to follow-up
14.	Hered 2007 [12]	6, M	orbit ND	ND	biopsy	**vincristine, etoposide, ifosfamide**[xiii] 5 months	partial response	R2 resection	3,5 years	NED
15.	Choi 2007 [13]	5, M	femur 5x3.8x2,5	no	biopsy	**vincristine, doxorubicin, cyclophosphamide**[xiv] 5 months	partial response	resection → CHT	6 months	D (cardiomyo pathy)
16.	Rekhi 2011 [14]	12, M	femur 6x4x3	no	biopsy	**vincristine, doxorubicin, cyclophosphamide** 2 months	stable disease	RTX → R0 resection	ND	ND
17.	Maroun 2016 [15]	4, F	mandible 6.9x4.4x3.3	ND	biopsy	**vincristine, dactinomycin, cyclophosphamide** [xv] 2,5 months	partial response	R0 resection	>2 years	NED
18.	Creytens 2017 [16]	3, M	forearm 3	no	biopsy	**ifosfamide, vincristine, dactinomycin, doxorubicin**[xvi] 4 months	partial response	R0 resection	28 months	NED
19.	Moreau (a) 2018 [17]	inborn, M	maxilla 4.7x4	no	biopsy	**cyclophosphamide, vincristine, etoposide, carboplatin, doxorubicin**[xvii] 3 months	progression → stable disease after last course	R2 resection	>2,5 years	NED
20.	Khemiri 2018 [18]	7, M	mandible 8x4	ND	biopsy	**ifosfamide, etoposide**[xviii] ≈ 5 months	partial response	resection	ND	ND
21.	Nicosia 2017 [19]	48, M	skull ND	yes, cervical LN	LN biopsy	**topotecan, cyclophosphamide, etoposide, ifosfamide, etoposide**[xix] 5 months	partial response	lymphadenectomy after 3 CHT courses, R2 resection after 5 CHT courses→ another R2 resection → CHT + RTX → parents refused treatment → progression	≈ 1 year	DOD
22.	current study, patient 3*	3, F	suboccipital area, spinal canal 4x4x3.1	no	biopsy	**vincristine, cyclophosphamide, doxorubicin**[xx] 1 month	stable disease	R2 resection → CHT → progression after 3 years	4,5 years	alive with disease
**PATIENTS TREATED WITH CHT ONLY**										

patient's no.	author, year of study	patient's age at diagnosis [mo], sex	primary tumor site and size [cm]	metastases at diagnosis	initial management	CHT (cytostatic drugs, duration)	response to CHT	further course of treatment	follow-up from the beginning of treatment	outcome
23.	Woessmann 2003 [20]	2, M	maxilla ND	ND	biopsy	**vincristine, ifosfamide, dactinomycin** → **vinblastin, ifosfamide/cyclophosphamide, etoposide, doxorubicin/liposomal daunorubicin**, **dactinomycin**[xxi] 10 months	first regimen → progression second regimen → subtotal tumor calcification	-	22 months	alive with stable disease
24.	Sailukar 2007 [21]	3, M	maxilla 8x6	ND	biopsy	**vincristine, cyclophosphamide, dactinomycin**[xxii]	stable disease	parents refused local treatment	ND	ND
25.	Moreau (b) 2018 [22]	4, ND	mandible 5.5	no	biopsy	**etoposide, carboplatin**	partial response	-	16 months	alive with stable disease

CHT = chemotherapy; CNS = central nervous system; D = died; DOD = died of disease; F = female; HSCT = hematopoietic stem cell transplantation; LN = lymph nodes; M = male; ND = no data; NED = no evidence of disease; R0 = macroscopically complete resection with microscopically negative margins; R1 = macroscopically complete resection with microscopically positive margins, R2 = macroscopically incomplete resection; RTX = radiotherapy
* = patients who underwent different types of CHT and have been included in the tables more than once

[i] **vincristine** (1,5mg/m2)**, lomustine** (15mg/m2), **methylprednisolone** (300mg/m2 x3)**, procarbazine** (75mg/m2)**, hydroxyurea** (1500mg/m2)**, cisplatin** (60mg/m2)**, cytosine arabinoside** (300mg/m2)**, cyclophosphamide** (300mg/m2) every 4-6 weeks

[ii] alternating courses

[iii] 2 courses

[iv] „alternate courses" every 21 days

[v] **vincristine** (0.05 mg/kg), **cyclophosphamide** (750 mg/m2 initially started in dose and then slowly increased), 8 courses every 3 weeks,

[vi] 2 courses

[vii] 1 course of **vincristine** (1.5 mg/m2), **cyclophosphamide** (600 mg/m2) and **pirarubicin** (30 mg/m2) followed by 5 courses of **vincristine** (1.5 mg/m2) , **cyclophosphamide** (900 mg/m2) and **carboplatin** (450 mg/m2)

[viii] weekly **vincristine** until week 6 and then 3 x weekly **vincristine** and 3 x weekly **cyclophosphamide** courses

[ix] alternating **doxorubicin/dactinomycin,** 9 courses

[x] 2 courses of **cisplatin, etoposide, vindesine** and 1 course of **vincristine, dacarbazine, ifosfamide;** details in case description

[xi] **vincristine** (day 1; 0.7 mg/m2), **cyclophosphamide** (days 1-7; 75 mg/m2), **doxorubicin** (day 8; 18 mg/m2), **vincristine** (day 8; 0.7 mg/m2), **cisplatin** (day 15; 40 mg/m2), **tenoposide** (day 17; 125 mg/m2), 3 courses

[xii] **vincristine** (0.05 mg/kg), **doxorubicin** (40 mg/m2 initially started in dose and then slowly increased), **cyclophosphamide** (750 mg/m2 initially started in dose and then slowly increased), 1 course every 3 weeks for 12 months

[xiii] 7 courses every 3 weeks

[xiv] **vincristine** (0.05 mg/kg), **doxorubicin** (40 mg/m2), **cyclophosphamide** (750 mg/m2), 6 courses every 3 weeks

[xv] **vincristine** (0.025 mg/kg/dose), **dactinomycin** (0.025 mg/kg/dose), **cyclophosphamide (**36 mg/kg/dose), 3 courses every 3 weeks

[xvi] RMS 2005 protocol (standard risk group, subgroup D); **doxorubicin** was added after patient reached 6 months of age

[xvii] 1 course of low-dose **cyclophosphamide** and **vincristine**, 2 courses of **etoposide** and **carboplatin,** 1 course of c**yclophosphamide, doxorubicin** and **vincristine**

[xviii] 6 courses of **ifosfamide** (100 mg/kg for 3 days) and **etoposide** (5 mg/kg for 3 days).

[xix] **topotecan** (days 1-5; 0.375 mg/m2) and **cyclophosphamide** (days 1-5; 125 mg/m2), alternating with **cyclophosphamide** (day 1; 4 g/m2) and **etoposide** (days 2-4; 200 mg/m2) or **ifosfamide** (days 1-3; 3 g/m2) and **etoposide** (days 1-3; 150 mg/m2), 5 courses

[xx] 2 courses, details in case description

[xxi] **vincristine, ifosfamide, dactinomycin** 2 courses → **vinblastin, ifosfamide/cyclophosphamide, etoposide, doxorubicin/liposomal daunorubicin** and **dactinomycin** 13 courses every 3 weeks

[xxii] 1 course

1. Cohen, B.H.; Handler, M.S.; DeVivo, D.C.; Garvin, J.H.; Hays, A.P.; Carmel, P. Central nervous system melanotic neuroectodermal tumor of infancy: Value of chemotherapy in management. *Neurology* **1988**, *38*, 163–164, doi:10.1212/wnl.38.1.163-a.

2. De Chiara, A.; Van Tornout, J.M.; Hachitanda, Y.; Ortega, J.A.; Shimada, H. Melanotic Neuroectodermal Tumor of Infancy. A Case Report of Paratesticular Primary with Lymph Node Involvment. *Am. J. Pediatr. Hematol. Oncol.* **1992**, *14*, 356–360.

3. Hoshino, S.; Takahashi, H.; Shimura, T.; Nakazawa, S.; Naito, Z.; Asano, G. Melanotic neuroectodermal tumor of infancy in the skull associated with high serum levels of catecholamine. *J. Neurosurg.* **1994**, *80*, 919–924, doi:10.3171/jns.1994.80.5.0919.

4. Patankar, T.; Prasad, S.; Goel, A.; Perumpillichira, J.; Desai, A.P. Malignant melanotic neuroectodermal tumour of infancy affecting the occipital squama. *J. Postgrad. Med.* **1998**, *44*, 73–75.

5. Kumari, T.P.; Venugopal, M.; Mathews, A.; Kusumakumary, P. Effectiveness of chemotherapy in melanotic neurectodermal tumor of infancy. *Pediatr. Hematol. Oncol.* **2005**, *22*, 199–206, doi:10.1080/08880010590921450.

6. Yoo, I.H.; Yum, S.K.; Oh, S.-J.J.; Kim, K.-M.M.; Jeong, D.C. Melanotic neuroectodermal tumor of infancy disseminated by a ventriculoperitoneal shunt and diagnosed from the inguinal sac. *J. Pediatr. Hematol. Oncol.* **2014**, *36*, 61–64, doi:10.1097/MPH.0b013e31829dd114.

7. Higashi, K.; Ogawa, T.; Onuma, M.; Usubuchi, H.; Imai, Y.; Takata, I.; Hidaka, H.; Watanabe, M.; Sasahara, Y.; Koyama, S.; et al. Clinicopathological features of melanotic neuroectodermal tumor of infancy: Report of two cases. *Auris Nasus Larynx* **2016**, *43*, 451–454, doi:10.1016/j.anl.2015.10.010.

8. Murphy, C.; Pears, J.; Kearns, G.J. Melanotic neuroectodermal tumour of infancy: surgical and chemotherapeutic management. *Ir. J. Med. Sci.* **2016**, *185*, 753–756, doi:10.1007/s11845-015-1323-4.

9. Azari, A.; Petrisor, D.; Wright, J.; Ghali, G.E. Metastatic Melanotic Neuroectodermal Tumor of Infancy: Report of a Case and Review of the Literature. *J. Oral Maxillofac. Surg.* **2016**, *74*, 2431–2440, doi:10.1016/j.joms.2016.05.028.

10. Pierre-Kahn, A.; Cinalli, G.; Lellouch-Tubiana, A.; Villarejo, F.J.; Sainte-Rose, C.; Pfister, A.; Couly, G. Melanotic Neuroectodermal Tumor of the Skull and Meninges in Infancy. *Pediatr. Neurosurg.* **1992**, *18*, 6–15.

11. Mello, R.J.V.; Vidal, A.K.L.; Fittipaldi, H.M.; Montenegro, L.T.; Calheiros, L.M.C.; Rocha, G.I. Melanotic Neuroectodermal Tumor of Infancy: Clinicopathologic Study of a Case, with Emphasis on the Chemotherapeutic Effects. *Int. J. Surg. Pathol.* **2000**, *8*, 247–251, doi:10.1177/106689690000800316.

12. Hered, R.W.; Smithwick IV, W.; Sandler, E.; Goldstein, J.D. Orbital melanotic neuroectodermal tumor of infancy successfully treated with chemotherapy and subtotal excision. *J. AAPOS* **2007**, *11*, 504–505, doi:10.1016/j.jaapos.2007.03.010.

13. Choi, I.S.; Kook, H.; Han, D.K.; Baek, H.J.; Jung, S.T.; Lee, J.H.; Park, J.G.; Hwang, T.J. Melanotic Neuroectodermal Tumor of Infancy in the Femur: A

Case Report and Review of the Literature. *J. Pediatr. Hematol. Oncol.* **2007**, *29*, 854–857.

14. Rekhi, B.; Suryavanshi, P.; Desai, S.; Gulia, A.; Desai, S.; Juvekar, S.L.; Puri, A.; Jambhekar, N.A. Melanotic neuroectodermal tumor of infancy in thigh of an infant-a rare case report with diagnostic implications. *Skeletal Radiol.* **2011**, *40*, 1079–1084, doi:10.1007/s00256-011-1113-7.

15. Maroun, C.; Khalifeh, I.; Alam, E.; Akl, P.A.; Saab, R.; Moukarbel, R. V. Mandibular melanotic neuroectodermal tumor of infancy: a role for neoadjuvant chemotherapy. *Eur. Arch. Oto-Rhino-Laryngology* **2016**, *273*, 4629–4635, doi:10.1007/s00405-016-4066-6.

16. Creytens, D.; Ferdinande, L.; Lecoutere, E.; Van Dorpe, J. Melanotic neuroectodermal tumour of infancy presenting as an undifferentiated round cell tumour in the soft tissue of the forearm. *Pathology* **2017**, *49*, 87–90, doi:10.1016/j.pathol.2016.10.006.

17. Moreau, A.; Galmiche, L.; Belhous, K.; Franchi, G.; Couloigner, V.; Nevoux, J.; Aymard, P.A.; Picard, A.; Minard-Colin, V.; Kadlub, N. Prenatal Diagnosis of a Melanotic Neuroectodermal Tumor of Infancy (MNTI): A Case Report with a Favorable Outcome after Chemotherapy Failure and Incomplete Resection. *J. Pediatr. Hematol. Oncol.* **2018**, *40*, 320–324, doi:10.1097/MPH.0000000000000982.

18. Khemiri, S.; Feki, J.; Khanfir, A.; Abdelmoula, M.; Frikha, M. Melanotic Neuroectodermal Tumor of Infancy: Presentation of a Case Affecting the Mandible. *Acta Med. Iran.* **2019**, *57*, 143–146, doi:10.18502/acta.v57i2.1772.

19. Nicosia, G.; Spennato, P.; Aliberti, F.; Cascone, D.; Quaglietta, L.; Errico, M.E.; Muto, M.; Ionna, F.; Cinalli, G. Giant melanotic neuroectodermal tumor of infancy (melanotic progonoma) of the head and neck: report of a malignant case. *J. Neurosurg. Pediatr.* **2017**, *19*, 538–545, doi:10.3171/2016.11.peds16509.

20. Woessmann, W.; Neugebauer, M.; Gossen, R.; Blütters-Sawatzki, R.; Reiter, A. Successful chemotherapy for melanotic neuroectodermal tumor of infancy in a baby [1]. *Med. Pediatr. Oncol.* **2003**, *40*, 198–199, doi:10.1002/mpo.10135.

21. Sailukar, M.; Bhagwat, R.; Seth, T. Melanocytic neuroectodermal tumor of infancy. *J. Indian Assoc. Pediatr. Surg.* **2007**, *12*, 136–137.

22. Moreau, A.; Galmiche, L.; Minard-Colin, V.; Rachwalski, M.; Belhous, K.; Orbach, D.; Joly, A.; Picard, A.; Kadlub, N. Melanotic neuroectodermal tumor of infancy (MNTI) of the head and neck: A French multicenter study. *J. Cranio-Maxillo-Facial Surg.* **2018**, *46*, 201–206, doi:10.1016/j.jcms.2017.12.001.
