# Supplementary material for: The Role of Chemotherapy in Management of Inoperable, Metastatic and/or Recurrent Melanotic Neuroectodermal Tumor of Infancy—Own Experience and Systematic Review
Source: Cancers (Basel). 2021 Jul 31;13(15):3872. doi: 10.3390/cancers13153872 (PMC8345623; doi:10.3390/cancers13153872)
Supplement: Supplementary file 1 [file cancers-13-03872-s001.zip › 30.07.21 MNTI supplementary table S2 recurrence treatment.pdf]

Table S2. Detailed data of the patients treated with chemotherapy in the recurrence of MNTI.

PATIENTS WITH RECURRENCE TREATED WITH ADJUVANT CHT

patient's no	author, year of study	patient's age at diagnosis [mo], sex	primary tumor site and size [cm]	metastases at diagnosis	initial management (before adjuvant CHT)	adjuvant CHT (cytostatic drugs, duration)	response to CHT	further course of the treatment	follow-up from beginning of treatment	outcome
1.	Blank 1980 [1]	0,5, F	maxilla 3x1,5	ND	resection → 1st recurrence → R2 resection	**vincristine, dactinomycin, cyclophosphamide**	complete response	-	13 months	NED
2.	Shokry 1986 [2]	2, M	maxilla 2,5	ND	resection → 1st recurrence → resection → 2nd recurrence → resection	**cyclophosphamide, doxorubicin**[i]	progression	progression → RTX	16 months	DOD
3.	Atkinson 1989 [3]	4, F	skull 4x4	ND	R1 resection → 1st recurrence → resection	**etoposide**	no recurrence	-	>1 year	NED
4.	Pierre-Kahn 1992* [4]	13, M	skull ND	ND	biopsy → resection → 1st recurrence with intracranial metastases → CHT → progression → resection	**cyclophosphamide**, **carboplatin** → **cyclophosphamide**, **etoposide**[ii]	progression	progression	ND	progression when lost to follow-up
5.	Shaia 2002 [5]	2, M	maxilla ND	ND	biopsy → resection → 1st recurrence → R1 resection	**carboplatin, cyclophosphamide, doxorubicin**, **etoposide**[iii] 5 months	no recurrence	-	1 year	NED
6.	Kumari 2005 [6]	4, M	orbit 2,6x1,8	ND	resection → 1st recurrence → R2 resection	**vincristine, cyclophosphamide, doxorubicin**[iv] 6 months	partial response	-	8 months	alive with disease, on treatment
7.	Haque 2012 [7]	2, M	maxilla ND	ND	resection → 1st recurrence → R2 resection	**cyclophosphamide, vincristine**[v] 2 months	stable disease	-	ND	alive with stable disease
8.	Rickart 2019 [8]	1,5, M	maxilla ND	ND	curettage→ curettage → 1st recurrence → R1 resection	**cyclophosphamide**, **vincristine**[vi] 1,5 months	no recurrence	-	17 years	NED
9.	Rickart 2019 [8]	1, M	maxilla ND	ND	curettage → 1st recurrence → R1 resection	**vincristine, cisplatin**, **etoposide**, **cyclophosphamide**, **carboplatin**[vii] 4 months	no recurrence	-	14 years	NED
10.	Rickart 2019 [8]	6, M	maxilla ND	ND	curettage → curettage → 1st recurrence → R1 resection	**vincristine, cisplatin**, **etoposide**, **cyclophosphamide**, **carboplatin**[viii] 4 months	no recurrence	-	>5 years	NED
11.	Pereira 2020 [9]	12, M	skull, 4.5×4.2×3.6	ND	R1 resection → 1st recurrence → resection → 2nd recurrence → R2 resection → R2 resection	**vincristine, doxorubicin, cyclophosphamide, carboplatin**[ix] ≈ 5 months	partial response	CHT[x] → progression with leptomeningeal dissemination → RTX	≈ 4 years	alive with disease
12.	current study, patient 2*	6, F	maxilla ND	no	R2 resection → 1st recurrence → R2 resection → 2nd recurrence → CHT → R1 resection	**vincristine, dactinomycin, cyclophosphamide**[xi] 5 months	no recurrence	-	6 years	ALL 7 mo after MNTI treatment

										CHT, allo-HSCT → complete response, NED

PATIENTS WITH RECURRENCE TREATED WITH NEOADJUVANT CHT

patient's no	author, year of study	patient's age at diagnosis [mo], sex	primary tumor site and size [cm]	metastases at diagnosis	initial management (before neoadjuvant CHT)	neoadjuvant CHT (cytostatic drugs, duration)	response to CHT	further course of the treatment	follow-up from beginning of treatment	outcome
13.	Dehner 1979 [10]	4, M	maxilla 2,5x2x1,5	ND	R1 resection → R0 resection → 1st recurrence → R1 resection → 2nd recurrence → R1 resection → 3rd recurrence with lymph nodes meta → RTX	**cyclophosphamide, methotrexate**, **vincristine**[xii]	progression	-	34 months	DOD
14.	Pierre-Kahn 1992* [4]	13, M	skull ND	ND	biopsy → resection → 1st recurrence with intracranial metastases	**cyclophosphamide**, **carboplatin**, **cyclophosphamide**, **etoposide** [xiii]	partial response (clear-up of metastases, local regression)	progression → resection → CHT → progression	ND	progression when lost to follow-up
15.	Neven 2008 [11]	4, M	mandible 3,5	ND	biopsy → resection → 1st recurrence → R1 resection →2nd recurrence → resection → 3rd recurrence	**vincristine**, **ifosfamide**, **carboplatin**, **etoposide**[xiv]	complete response	-	14 years	NED
16.	Enriquez 2011 [12]	2, M	maxilla 3.8x3x2	ND	resection → 1st recurrence	**cyclophosphamide, vincristine, doxorubicin**[xv]	partial response	resection	≈ 6 months	NED
17.	Davis 2015 [13]	2, M	maxilla 1,5x1	ND	biopsy → R0 resection → 1st recurrence	**vincristine**, **cyclophosphamide**, **doxorubicin**[xvi]	progression	R0 resection	≈ 2,5 years	NED
18.	current study, patient 1	2, F	maxilla 2,3x2,2x 1,8	no	R2 resection → 1st recurrence	**cyclophosphamide, vincristine, doxorubicin, etoposide, carboplatin, ifosfamide**[xvii] 5 months	partial response	R0 resection	5,5 years	NED
19.	current study, patient 2*	6, F	maxilla ND	no	R2 resection → 1st recurrence → R2 resection → 2nd recurrence	**vincristine, dactinomycin, cyclophosphamide**[xviii] 5 months	partial response	R1 resection → CHT → complete response	6 years	ALL 7 mo after MNTI treatment CHT, allo-HSCT → complete response, NED
20.	current study, patient 4	4, F	maxilla 1x1	no	R1 resection → 1st recurrence	**vincristine, cyclophosphamide, doxorubicin**[xix] 3 months	stable disease	R2 resection → stable disease	2 years	alive with stable disease

CHT = chemotherapy; DOD = died of disease; F = female; HSCT = hematopoietic stem cell transplantation; M= male; ND = no data; NED = no evidence of disease; R0 = macroscopically complete resection with microscopically negative margins; R1 = macroscopically complete resection with microscopically positive margins; R2 = macroscopically incomplete resection; RTX = radiotherapy;
* = patients who underwent different types of CHT and have been included in the tables more than once

[i] 10 courses, also “various other chemotherapeutic drugs”
[ii] **cyclophosphamide** (100mg/m2/day), **carboplatin** (100mg/m2/day) → **cyclophosphamide** (150mg/m2/day), **etoposide** (1g/m2/day)
[iii] 8 courses
[iv] **vincristine** (0.05 mg/kg), **cyclophosphamide** (750 mg/m2; initially started in dose and then slowly increased), **doxorubicin** added after 3 months (40 mg/m2; initially started in dose and then slowly increased)
[v] 3 courses
[vi] 2 courses
[vii] 6 courses
[viii] 6 courses
[ix] 6 courses with a 21-day interval. 1 course: **vincristine** (1.5 mg/m2), **doxorubicin** (30 mg/m2), **cyclophosphamide** (600 mg/m2/day); 2-6 course: **cyclophosphamide** (900 mg/m2), **vincristine** (1.5 mg/m2), **carboplatin** (450 mg/m2)
[x] undefined CHT
[xi] 8 courses
[xii] **cyclophosphamide** (25 mg/kg in 5 doses) → “high-dose” **methotrexate** (690 mg/m2), **vincristine** (1.5 mg/m2)
[xiii] **cyclophosphamide** (100mg/m2/day), **carboplatin** (100mg/m2/day) → **cyclophosphamide** (150mg/m2/day), **etoposide** (1g/m2/day)
[xiv] 6 courses of VECI neuroblastoma chemotherapy protocol: **vincristine** day 1, **ifosfamide** days 1 and 2, **carboplatin** day 3, and **etoposide** day 4
[xv] 6 courses
[xvi] **vincristine** weekly, **cyclophosphamide** every other week, after 3 courses **doxorubicin** added
[xvii] **CADO** (cyclophosphamide, vincristine, doxorubicin) 1 course → **VP-16+Carbo** (etoposide + carboplatin) 2 courses → **I2VAdr** (ifosfamide, vincristine, doxorubicin) 1 course
→ **I2VE** (ifosfamide, vincristine, etoposide) 1 course → **CADO** (cyclophosphamide, vincristine, doxorubicin) 1 course → **VP-16+Carbo** (etoposide + carboplatin) 1 course → **CO** (vincristine, cyclophosphamide) 1 course
[xviii] 6 courses
[xix] 4 courses

1. Blank, E.; Runckel, D.N. Case Report 119. *Skeletal Radiol.* **1980**, *5*, 179–182, doi:10.1148/radiology.160.2.452.
2. Shokry, Al.; Briner, J.; Makek, M. Malignant Melanotic Neuroectodermal Tumor of Infancy: A Case Report. *Pediatr. Pathol.* **1986**, *5*, 217–223, doi:10.1007/BF00570263.
3. Atkinson Jr, G.O.; Davis, P.C.; Patrick, L.E.; Winn, K.J.; Ball, T.I.; Wyly, J.B. Melanotic neuroectodermal tumor of infancy. MR findings and a review of the literature. *Pediatr. Radiol.* **1989**, *20*, 20–22.
4. Pierre-Kahn, A.; Cinalli, G.; Lellouch-Tubiana, A.; Villarejo, F.J.; Sainte-Rose, C.; Pfister, A.; Couly, G. Melanotic Neuroectodermal Tumor of the Skull and Meninges in Infancy. *Pediatr. Neurosurg.* **1992**, *18*, 6–15.
5. Shaia, W.T.; DiNardo, L.J.; Underhill, T.E.; Cesca, C.E. Recurrent melanotic neuroectodermal tumor of infancy. *Am. J. Otolaryngol.* **2002**, *23*, 249–252, doi:10.1053/ajot.2002.123463.
6. Kumari, T.P.; Venugopal, M.; Mathews, A.; Kusumakumary, P. Effectiveness of chemotherapy in melanotic neurectodermal tumor of infancy. *Pediatr. Hematol. Oncol.* **2005**, *22*, 199–206, doi:10.1080/08880010590921450.
7. Haque, S.; McCarville, M.B.; Sebire, N.; McHugh, K. Melanotic neuroectodermal tumour of infancy: CT and MR findings. *Pediatr. Radiol.* **2012**, *42*, 699–705, doi:10.1007/s00247-011-2339-1.
8. Rickart, A.J.; Drummond-Hay, V.; Suchak, A.; Sadiq, Z.; Sebire, N.J.; Slater, O.; Mills, C. Melanotic neuroectodermal tumour of infancy: Refining the surgical approach. *Int. J. Oral Maxillofac. Surg.* **2019**, *48*, 1307–1312, doi:10.1016/j.ijom.2019.02.011.
9. Pereira, A.A.C.; de Jesus Rozante, M.M.; Bauer Doveinis, R.; Porcelli Salvarani, C.; Hissa Anegawa, T.; da Costa Souza, P.; Brat, D.J.; de Oliveira Borges, A.C. The recurrence of the melanotic neuroectodermal tumour of infancy: an unusual presentation of a rare tumour. *Ecancermedicalscience* **2020**, *14*, doi:10.3332/ecancer.2020.1049.
10. Dehner, L.P.; Sibley, R.K.; Sauk, J.J.; Vickers, R.A.; Nesbit, M.E.; Leonard, A.S.; Waite, D.E.; Neeley, J.E.; Ophoven, J. Malignant Melanotic Neuroectodermal Tumor of Infancy. A Clinical, Pathologic, Ultrastructural and Tissue Culture Study. *Cancer* **1979**, *43*, 1389–1410.
11. Neven, J.; Hulsbergen-van der Kaa, C.; Groot-Loonen, J.; de Wilde, P.C.M.; Merkx, M.A.W. Recurrent melanotic neuroectodermal tumor of infancy: a proposal for treatment protocol with surgery and adjuvant chemotherapy. *Oral Surgery, Oral Med. Oral Pathol. Oral Radiol. Endod.* **2008**, *106*, 493–496, doi:10.1016/j.tripleo.2008.02.001.
12. Enriquez, A.M.; Carnate Jr, J.M. Melanotic neuroectodermal tumor of infancy. *Philipp. J. Otolaryngol. Neck Surg.* **2011**, *26*, 51–54, doi:10.5858/arpa.2018-0241-RA.
13. Davis, J.M.; DeBenedictis, M.; Frank, D.K.; Lessin, M.E. Melanotic Neuroectodermal Tumor of Infancy: A Wolf in Sheep's Clothing. *Ann. Otol. Rhinol.*

Laryngol. **2015**, *124*, 97–101, doi:10.1177/0003489414543070.
