# Supplementary material for: The Role of Chemotherapy in Management of Inoperable, Metastatic and/or Recurrent Melanotic Neuroectodermal Tumor of Infancy—Own Experience and Systematic Review
Source: Cancers (Basel). 2021 Jul 31;13(15):3872. doi: 10.3390/cancers13153872 (PMC8345623; doi:10.3390/cancers13153872)
Supplement: Supplementary file 1 [file cancers-13-03872-s001.zip › 30.07.21 Supplementary Material 3 - Systematic literature search protocol.pdf]

Systematic Review: The role of chemotherapy in management of inoperable, metastatic and/or recurrent melanotic neuroectodermal tumor of infancy – own experience and systematic review.

Date of the literature search: 26.04.2021

SYSTEMATIC LITERATURE SEARCH

1. PUBMed/Medline database:

melanotic neuroectodermal tumor OR	AND	chemotherapy OR
retinal anlage tumor OR		systemic treatment
melanotic progonoma		

no.	search terms	results
#1	((melanotic neuroectodermal tumor) OR (melanotic progonoma)) OR (retinal anlage tumor)	492
#2	(chemotherapy) OR (systemic treatment)	3,656,927
#3	#1 AND #2	**42**

2. SCOPUS

melanotic neuroectodermal tumor OR	AND	chemotherapy OR
retinal anlage tumor OR		systemic treatment
melanotic progonoma		

no.	search terms	results
#1	((“melanotic neuroectodermal tumor”) OR (“melanotic progonoma”)) OR (“retinal anlage tumor”)	885
#2	(chemotherapy) OR (“systemic treatment”)	2,141,128
#3	#1 AND #2	**209**

3. Web of Science

melanotic neuroectodermal tumor OR	AND	chemotherapy OR
retinal anlage tumor OR		systemic treatment
melanotic progonoma		

no.	search terms	results

#1	ALL=”melanotic neuroectodermal tumor” OR ALL=”retinal anlage tumor” OR ALL=”melanotic progonoma”	329
#2	ALL=chemotherapy OR ALL=”systemic treatment”	583,815
#3	#1 AND #2	**40**

42 + 209 + 40 = 291 papers

After automatic (Mendeley) removal of duplicates: 227 papers

After manual removal of duplicates: 215 papers

After abstract reading: 90 papers

Reports not retrieved: (n=1)
No use of chemotherapy in MNTI reported (n = 57)
Unclear histology (n = 3)
No information on cytostatic drugs used (n = 1)
No information on the response to chemotherapy (n = 2)
Concomitant use of radiotherapy (n=1)

After full-text reading: 25 papers

ADDITIONAL CITATION SEARCH

Publications found in the references of other manuscripts (n=12)

Duplicated case (n = 1)
Unclear histology (n = 1)
No information on cytostatic drugs used (n = 2)

After full-text reading: 8 papers

ALL STUDIES INCLUDED IN REVIEW:

All studies found via systematic search and via reference review (n = 33) with (n = 43) individual patients (n=5 excluded due to missing data/duplication).

Additional patients from authors’ institutions (n = 4)

Individual patients included in review: 42 patients
